# Supplementary figures and images for: Cardiovirus leader proteins retarget RSK kinases toward alternative substrates to perturb nucleocytoplasmic traffic
Source: PLoS Pathog. 2022 Dec 12;18(12):e1011042. doi: 10.1371/journal.ppat.1011042 (PMC9779665; doi:10.1371/journal.ppat.1011042)

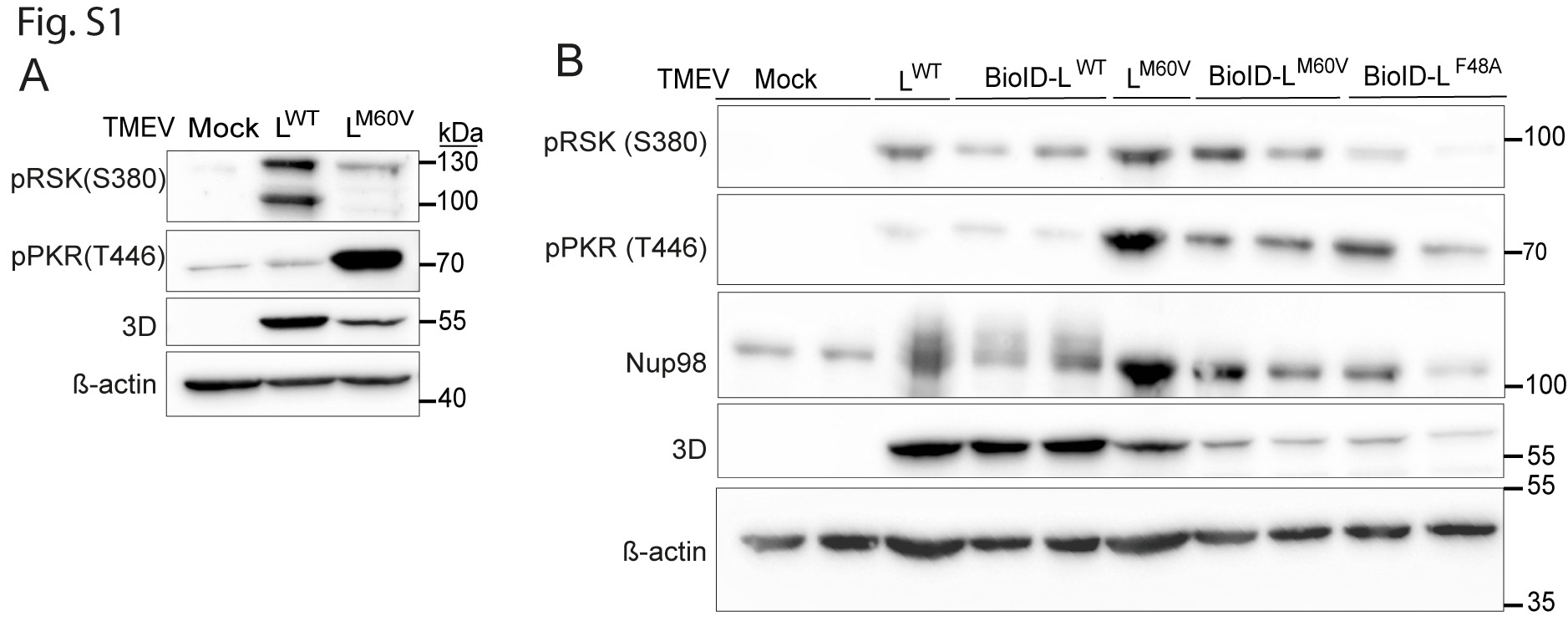

Supplement: S1 Fig — (A) L protein activities are conserved in HeLa BioID-RSK cells. HeLa BioID-RSK cells were infected with LWT and LM60V viruses for 16h. Western blot showing BioID-RSK activation by the LWT and LM60V proteins (p-RSK at S380) and PKR inhibition by LWT but not by LM60V (p-PKR at T446 is a marker of PKR activation). (B) BioID-L fusion proteins maintain their corresponding activities. Immunoblots show the detection of activated RSK (p-S380), activated PKR (p-T446) and Nup98 hyperphosphorylation (migration shift) in HeLa cells infected for 16h with BioID-LWT, BioID-LM60V, BioID-LF48A replicons (2 lanes each) and, as a control, with LWT and LM60V viruses (1 lane each). Viral polymerase 3D and ß-actin were detected as infection and loading controls respectively. (TIF) [file ppat.1011042.s001.tif]

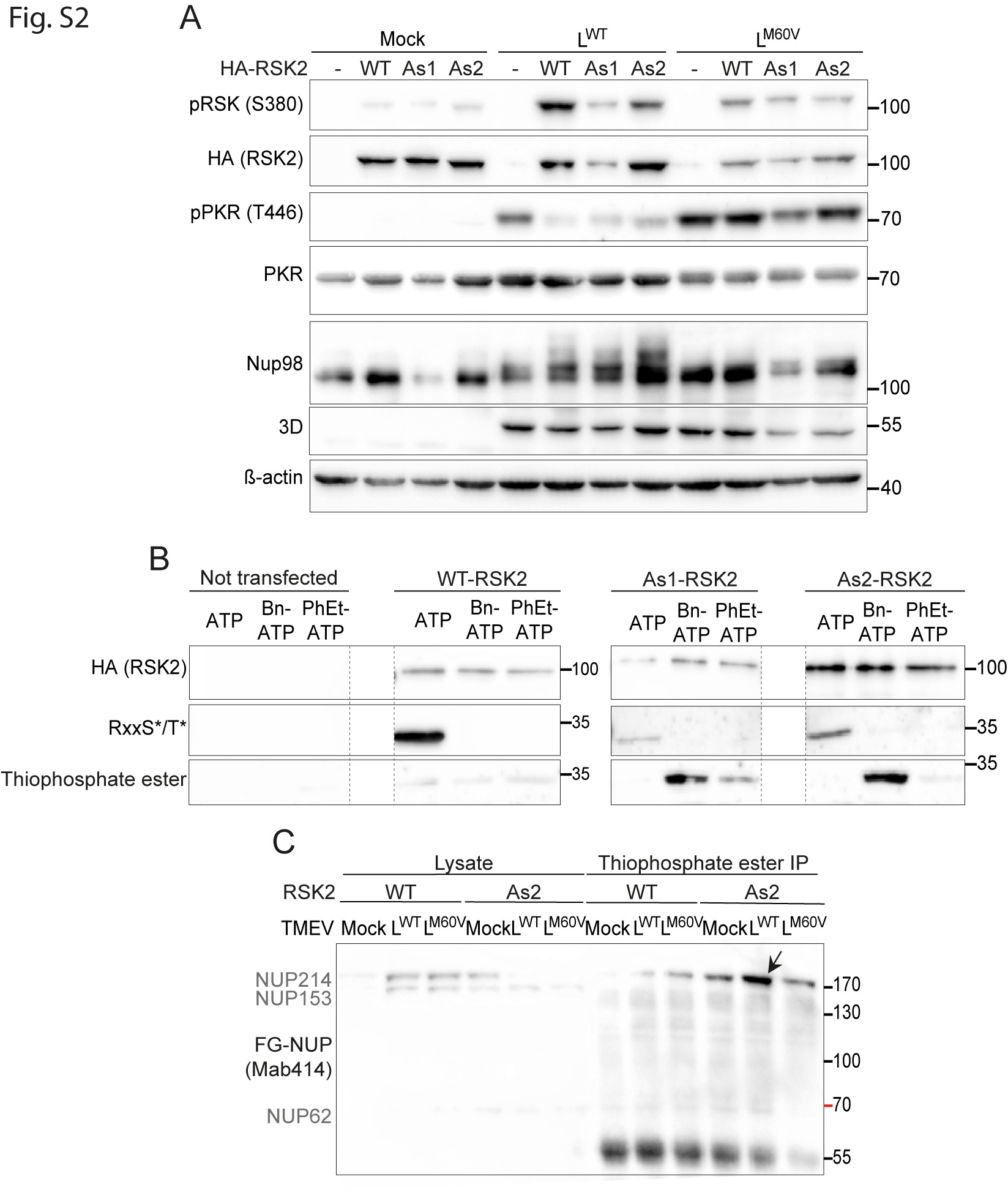

Supplement: S2 Fig — (A) Analog-sensitive RSKs (As1 and As2-RSKs) recue L protein activities. HeLa RSK-TKO cells transduced with an empty vector (-) or with lentiviral vectors expressing WT-RSK, As1-RSK or As2-RSK, were infected (MOI 2.5) for 15h with LWT or LM60V viruses. Western blots show the detection of HA-(RSK), P-PKR (T446), PKR, NUP98, 3D viral polymerase as a control of infection and ß-actin as a loading control. RSKs activation (p-RSK S380) PKR inhibition (inhibition of pPKR T446) and NUP98 hyperphosphorylation (shift upwards) in As-RSK expressing cells paralleled those observed in WT-RSK expressing cells. (B) GST-S6 (thio)-phosphorylation by As1, As2 and WT RSKs in an in vitro kinase assay. 293T cells were transfected with plasmids coding for WT-RSK, as1-RSK or as2-RSK. 6 hours post-transfection, cells were treated with phorbol myristate acetate (PMA) to activate RSKs. 18 hours later, RSKs were immunoprecipitated with an anti-HA antibody. An in vitro kinase assay was performed with the immunoprecipitated RSKs, GST-S6 (recombinant substrate) and either ATP or N6-Bn-ATP-γ-S or N6-PhEt-ATP-γ-S. Reaction proceeded for 30min at 30°C before alkylation by PNBM for 2h at room temperature. Reaction was stopped by addition of sample buffer. Samples were analyzed by western blot with antibodies against HA-(RSK), RxxS*/T* (antibody against phosphorylated RSK substrates; here: phospho-GST-S6), and against thiophosphate ester. WT RSK was able to use ATP but was not able to use the ATP analogs to phosphorylate GST-S6 (RxxS*/T*). As1 and As2-RSK were able to use ATP to phosphorylate GST-S6 (RxxS*/T*) but were also able to use both ATP analogs, with a preference for N6-Bn-ATP-γ-S (Thiophosphate ester). Dashed lines between lanes indicate deletion of irrelevant lanes from the same membrane. (C) Thiophosphorylation of NUP214 by As2-RSK. Immunoblots showing NUP214 in the thiophosphate ester IP fraction when LWT is present. HeLa cells expressing As2-RSK or WT-RSK were infected with TMEV for 8h (M [file ppat.1011042.s002.tif]
