# Supplementary material for: Cardiovirus leader proteins retarget RSK kinases toward alternative substrates to perturb nucleocytoplasmic traffic
Source: PLoS Pathog. 2022 Dec 12;18(12):e1011042. doi: 10.1371/journal.ppat.1011042 (PMC9779665; doi:10.1371/journal.ppat.1011042)
Supplement: Attachment — Submitted filename: Lizcano-Perret response to reviewers.pdf [file ppat.1011042.s003.pdf]

Response to reviewers

PPATHOGENS-D-22-01122

Part I - Summary

Please use this section to discuss strengths/weaknesses of study, novelty/significance, general execution and scholarship.

Reviewer #1: The manuscript by Lizcano-Perret et al describes a novel mechanism by which viruses promote their replication and reduce antiviral immunity, namely via proteins that act as adaptor proteins to retarget host cell kinases towards specific substrates. As an example of this 'model of the clamp', they show that cardioviruses of the family picornaviridae code for non-structural proteins (Leader proteins) that can clamp both the host protein kinase RSK and a host substrate, nucleoporins. This enforces hyperphosphorylation of the nucleoporins, thereby triggering nucleocytoplasmic trafficking perturbation, which supports viral replication. The study encompasses several high-end methodologies to dissect the role of host and virus proteins in this process, such as a reciprocal BioID proxeosome determination to identify substrates binding to the Leader-RSK complex and an analog-sensitive kinase system. The data are convincing and the manuscript is well written. I have a few requests for further clarification, which I specify below.

Reviewer #2: This study examines the mechanisms governing ribosomal S6 kinase (RSK) activity and substrate selection in cardiovirus-infected cells. TMEV expressing wild type L protein disrupts nucleo-cytoplasmic trafficking in HeLa cells as evidenced by relocalization of PTB, GFP-NES and RFP-NLS and phosphorylation of Nup98. Viruses expressing a mutant L protein (M60V) lose this ability. KO of RSK prevents relocalization and Nup98 phosphorylation and this can be rescued by expression of any RSK isoform. Similar results are obtained with EMCV expressing WT L or L with a mutant zinc finger domain. Fusion of a BioID tag to RSK, WT L or M60V L is used to identify proteins that interact with L protein and are substrates for the RSK. Immunoprecipitation and mass spec analysis of biotinylated proteins reveals significant binding to FG-Nups, particularly Nup98 and this is reduced in cells expressing M60V. Confocal microscopy reveals that biotin labeled proteins are at the nuclear rim, but only if Lwt is also expressed. To identify RSK substrates, a mutant form of RSK is generated that utilizes N6-alkylated ATP-gamma-S for phosphorylation. Immunopercipitation of Nup98 revealed the presence of thiophosphorylated forms in cells infected with TMEV expressing WT L but not M60V, suggesting that Nup98 is an L-dependent substrate for RSK in infected cells.

This is a well done study that significantly adds to our understanding of how L modulates the kinase activity of RSK in infected cells. Convincing data is presented that L serves as a scaffold for binding to RSK and substrates, such as FG-Nups, so that they can be phosphorylated. The work suggests a model where RSK bound to L is targeted to the nuclear pore complex via binding of L to FG-Nups, where it phosphorylates these proteins and disrupts trafficking between the nucleus and cytoplasm. Given that other viruses and even bacteria also usurp RSK activity in cells, these findings are broadly relevant to those studying host-pathogen interactions.

Reviewer #3: The Cardiovirus leader proteins retarget RSK kinases toward alternative substrates to perturb nucleocytoplasmic traffic manuscript by Belé Lizcano-Perret, et. al conveys that p90-RSKs can be recruited to a given substrate by Cardiovirus leader proteins. The authors use Bio-ID to determine RSK binding partners as a novel strategy and show that RSK phosphorylation of nuclear pore proteins disrupts nuclear trafficking by microscopy. However, additional controls are required to adequately interpret data in the manuscript.

> We thank the reviewers for their positive and constructing comments. We did a series of experiments and amended the manuscript to take almost all of their comments into account. Below is a point-by-point response to the specific comments.

Part II – Major Issues: Key Experiments Required for Acceptance

Reviewer #1: Figure 6 and 7:

A more quantitative image analysis approach should be conducted to prove that the proteins biotinylated by BioID-RSK colocalize with the nuclear membrane/nucleoporins (eg by showing profile intensity plots (intensity vs distance plots) overlaid for both biotinylated proteins and nucleoporin fluorescence signals). This should also clarify that it seems that there is similar co-localization for WT-L and M60V-L, e.g. that there are quantitative differences.

> We fully agree with this Reviewer that intensity vs distance plots help to interpret the data (which looked clear to us, having seen so many pictures). Such plots have been added in revised Figs 7 and 8.

The authors should also explain why for Fig 6 the costaining is with NUP98, while in Fig 7 it is with POM121.

> We formerly used NUP98 (mouse antibody) in one experiment and POM121 (rabbit antibody) in another experiment, for technical reasons, to optimize the use of secondary antibodies in multiple stainings. We now repeated some experiments in which we costained and show the colocalization of NUP98 and POM121 (revised Fig. 7C).

Fig 6:

As additional proof for Leader-driven localization of RSK to the nuclear membrane, the authors should show the co-localization of biotinylated proteins for the condition with F48A-L (RSK substrate biotinylation without Leader binding).

> We agree that this additional experiment would help to convince about the relocalization of RSK by L. We thus performed a new series of infection experiments to include infection of BioID-RSK expressing cells by the F48A mutant virus. Data shown in revised Fig. 7 confirm the absence of biotinylation of nuclear envelope proteins when L cannot interact either with NUPs (L^{M60V}) or with RSK (L^{F48A}).

Reviewer #2: (No Response)

Reviewer #3:

• Microscopy images demonstrating changes in nuclear-cytoplasmic diffusion (Fig 2A, Fig 2C, Fig 2E, Fig 2G, and Fig 3A, Fig 3C, Fig 3E) are difficult to interpret. Confocal microscopy eliminates out-of-plane signal, so results may not adequately capture all diffused protein, thereby skewing results. This is particularly problematic for images shown in Fig 3, which are shown without an accompanying nuclear stain. 3D reconstruction or markers are required to control for variation in imaging height in confocal microscopy.

> Additional experiments were performed on live cells to gather 3D reconstruction images showing (or not) diffusion of GFP-NES in the nucleus (with Hoechst staining). These data are presented in revised Fig. 4

• Furthermore, accompanying quantitation of protein diffusion is inadequate. To provide a percent count of diffusion-positive cells, at least 100 infected cells per field should be evaluated and quantitative cutoffs for positive and negative cells should be provided. As an alternative, authors could quantitate fluorescence to present the nuclear to cytoplasmic ratio of protein localization.

> The counts of diffusion-positive cells were done on the merge images showing both GFP-NES and RFP-NLS. To present more accurately the counting strategy, the following sentence was added in

legend to Fig. 3; "Focus was set on the red channel and diffusion of GFP-NES was evaluated on merge images. 50 cells per experiment (n=4) were counted and considered diffusion-positive when green fluorescence was as prominent as red fluorescence in the nucleus."
We honestly reported the number of cells that were counted and do not agree that at least 100 infected cells per field are required to provide a percent count. At the magnification used, fields usually contained less than 50 cells.

• Experiments utilizing RSK knockout and overexpression require additional controls for interpretation. In Figs 2C-D, Figs 2G-H, and Figs 3A-C, authors knockout or overexpress RSK isoforms. Expression levels of RSK isoforms should be shown to demonstrate efficacy of knockout and overexpression. Furthermore, the effect of RSK knockout or overexpression on TMEV replication is not apparent, as differences in 3D expression shown in Fig 2D are marginal. Results should be quantitated.

> Blots were added in revised Fig. 2D showing HA (HA-RSK) and RSK (mostly RSK1-specific) detection, showing that RSK1, -2, -3 and -4 are re-expressed at different level. RSK2 and -3 re-expression is less intense and cannot be considered as overexpressed.
On the other hand, viral replication was not the focus of this experiment, we do not think that adding quantitative 3D blots would add to the interpretation of the experiment. 3D detection is shown to confirm infection of the samples.

• Results demonstrating localization of Bio-ID-RSK targets at the nuclear periphery shown in Figs 6 and 7 are not convincing. Nuclear fluorescence from biotinylated proteins is too high to evaluate small changes on the nuclear periphery. Selective clearing to reduce fluorescence within the nucleus would aid in evaluation of the nuclear periphery. As above, quantitative criteria for positive and negative cells should be provided and more cells should be evaluated per sample.

> As recommended by Reviewer #1, we added intensity vs distance plots in the revised version (revised Figs 7 and 8) to provide a more quantitative view of the fluorescence associated with the nuclear envelope.
On the other hand, we do not believe that doing more experiments (counting more cells) is needed given the reproductibility of the experiment and given the black-and-white differences observed between constructs.

• Additional controls are required to evaluate results shown in Fig 8. The Shokat method has not been reported for RSK2, and it is unknown whether mutation of the RSK2 ATP binding pocket changes the RSK2 substrate repertoire. Additional controls are required to demonstrate that RSK2 substrate binding is not altered.

> We agree that the question as whether the mutation of the ATP binding pocket might alter RSK substrate specificity is an interesting one.
1. We are currently using a proteomics approach to analyze the substrate repertoire of analog-sentive RSK1. Our data suggest no alteration in the phosphorylation consensus sequence of as-RSK1 versus the reported consensus for RSK1, suggesting that modification of the ATP binding pocket has little if any influence on substrate specificity (work in progress).
2. Our experiments compared RSK substrates for the same as-RSK (as-RSK2) in cells infected with different viruses. Therefore, substrate repertoire differences observed between the same as-RSK expressing cells infected with L^{WT} and L^{M60V} viruses should not be biased by the mutation introduced in RSK to accommodate the ATP analog.

We added a paragraph in the discussion (lines 450-57 of the revised manuscript)

• Statistical methods used require clarification. One-way ANOVA cannot be used to evaluate

statistical differences between individual groups, yet p-values between experimental groups are reported in multiple graphs. Post-hoc tests are required to make individual comparisons when more than two groups are evaluated.

> We corrected the figure legends to explain that multiple comparisons were made but that the significance of only the most relevant comparisons was shown. In addition, the materials and methods section was completed to mention that Tukey's multiple comparisons tests were performed.

Part III – Minor Issues: Editorial and Data Presentation Modifications
Please use this section for editorial suggestions as well as relatively minor modifications of existing data that would enhance clarity.

Reviewer #1: Table in fig 5B:
please provide insight in why the BioID-RSK experiments did not yield any FG-NUP hit that reached statistical significance in the fold change L-WT over L-M60V, whereas several of the NUPs reach significant p-values in the BioID-L experiments. What does this say about the methodology, or about how the RSK-Leader complex could bind substrates?
In connection to the above question, can the authors provide potential explanations for why NUP62 and NUP214 show a much higher abundance ratios in BioID-L than in BioID-RSK?

Reviewer #2: One question that I think merits comment is why the BioID-RSK experiments didn't show as significant an association with the FG-Nups as the BioID-L and M60V experiments did.

Reviewer #3: (No Response)

> The next sentence was added in the manuscript (lines 276-278) to clarify the data:
"Lower significance exhibited by BioID-RSK compared with the BioID-L data likely reflects the fact that only part of the BioID-RSK molecules are retargeted by L toward new partners."
This difference is also illustrated by the differences seen with the fluo-streptavidin stainings shown in Figs 7 and 8 where the envelope localization is better visible in the case of BioID-L.
